# Targeted delivery of TGF-β mRNA to lung parenchyma using one-component ionizable amphiphilic Janus Dendrimers

**DOI:** 10.21203/rs.3.rs-4656663/v1

**Published:** 2024-07-12

**Authors:** Elena Atochina-Vasserman, Jaclynn Meshanni, Emily Stevenson, Dapeng Zhang, Rachel Sun, Nathan Ona, Erin Reagan, Elena Abramova, Chang-Jiang Guo, Melissa Wilkinson, Ishana Baboo, Yuzi Yang, Liuyan Pan, Devendra Maurya, Virgil Percec, Yongsheng Li, Andrew Gow, Drew Weissman

**Affiliations:** Perelman School of Medicine, University of Pennsylvania; University of Pennsylvania; Rutgers University; East China University of Science and Technology; Rutgers University; University of Pennsylvania Perelman School of Medicine; University of Pennsylvania Perelman School of Medicine; Rutgers University; Rutgers University; Rutgers University; University of Pennsylvania Perelman School of Medicine; East China University of Science and Technology; East China University of Science and Technology; Roy & Diana Vagelos Laboratories, Department of Chemistry, University of Pennsylvania; University of Pennsylvania; East China University of Science and Technology; Rutgers University; University of Pennsylvania

**Keywords:** one-component ionizable amphiphilic Janus Dendrimers, mRNA targeted delivery, anti-inflammatory cytokine, TGF-β, bleomycin injury, respiratory disease, lung parenchyma

## Abstract

Current clinical strategies for the delivery of pulmonary therapeutics to the lung are primarily targeted to the upper portions of the airways. However, targeted delivery to the lower regions of the lung is necessary for the treatment of parenchymal lung injury and disease. Here, we have developed an mRNA therapeutic for the lower lung using one-component Ionizable Amphiphilic Janus Dendrimers (IAJDs) as a delivery vehicle. We deliver an anti-inflammatory cytokine mRNA, transforming growth factor-beta (TGF-β), to produce transient protein expression in the lower regions of the lung. This study highlights IAJD’s potential for precise, effective, and safe delivery of TGF-β mRNA to the lung. This delivery system offers a promising approach for targeting therapeutics to the specific tissues, a strategy necessary to fill the current clinical gap in treating parenchymal lung injury and disease.

## INTRODUCTION

Acute lung injury (ALI) is a prevalent condition in the United States, with 200,000 new diagnoses each year, resulting in high morbidity and mortality (1–4). Injury progression is a result of epithelial barrier dysfunction, alveolar damage, pulmonary edema, and surfactant dysfunction brought on by changes in innate immune, epithelial, and endothelial cell dysfunction (1, 5–8). Many of the effects of ALI are observed in the lung parenchyma beyond the 16th generation. However, drug delivery to this area is challenging often leading to poor specificity and an uneven distribution (9–13). Therefore, there is a therapeutic gap in the treatment of ALI requiring the development of methods to deliver pharmacological agents to the lower lung. This is a particular challenge when administering a complex biologic, such as anti-inflammatory cytokine mRNA, to the site of injury.

Since the beginning of the severe acute respiratory syndrome coronavirus-2 (SARS-CoV2) pandemic in 2019, there have been a series of major breakthroughs in the development of nucleoside-modified mRNA vaccines by both major research institutions and the pharmaceutical industry (14–17). Currently, the leading delivery system for mRNA vaccines are four-component lipid nanoparticle (LNP) synthetic delivery systems, which consist of ionizable lipids, phospholipids, cholesterol, and polyethylene glycol (PEG)-conjugated lipids, which can be assembled by microfluidic or T-tube technology (18, 19). Despite their wide adoption for Covid-19 vaccinations, the targeted delivery of mRNA to the lung with four-component LNPs has not proven successful (20–26).

Recently, we reported a novel self-assembling one-component multifunctional sequence-defined ionizable amphiphilic Janus dendrimer (IAJD) synthetic delivery system for mRNA, consisting of functional hydrophilic dendrons conjugated to hydrophobic dendrons (27, 28). IAJDs and mRNA can be co-assembled into unilamellar and multilamellar onion-like dendrimersome nanoparticles (DNPs) by simple mixing in acetate buffer rather than microfluidic and T-tube devices. Previously we have shown that certain of these IAJDs display organ specificity (28). In addition to eliminating structural issues that can arise from mixtures of four-component LNPs, this technology may allow for programmed delivery to specific organs, such as the lung.

In this study, we explore the potential of one-component IAJDs to deliver therapeutic mRNA encoding the anti-inflammatory cytokine, transforming growth factor-beta (TGF-β) to the lung parenchyma. Additionally, we test the nontoxic and transient nature of its delivery, as persistent TGF-β delivery may result in excessive pro-resolution signaling, leading to the development of lung fibrosis over time (29–32). Here, we confirm that IAJDs can be utilized for the successful and targeted delivery of cytokine mRNA diffusely throughout the lung and that the effects of this delivery are transient, reducing the risk of fibrotic development. Furthermore, we evaluate the potential for IAJD targeted delivery of TGF-β mRNA to limit ALI using an intratracheal bleomycin (ITB) model (3, 32, 33). These studies contribute to the progress of genetic nanomedicine and raise the possibility of lung-based nanotherapeutics.

## METHODS

### mRNA production and characterization

Codon-optimized sequences of firefly luciferase (Luc-mRNA, GenBank: MK484108.1) or tumor growth factor-β (TGFβ-mRNA, GenBank: NM_011577.2) were synthesized and cloned into an mRNA production plasmid as previously described (24, 34). Briefly, nucleoside-modified mRNAs were transcribed to contain 101 nucleotide-long poly(A) tails. mRNAs were modified with m1Ψ–5′-triphosphate (TriLink, # N-1081) instead of UTP and capped cotranscriptionally using the trinucleotide cap1 analog, CleanCap (TriLink, # N-7413). mRNA was purified by cellulose (Sigma-Aldrich, # 11363–250G) purification (24). All mRNA were analyzed for quality control through agarose gel electrophoresis, dsRNA, endotoxin and IFN-α assays and in vitro transfection. mRNA was stored at − 20°C until ready for use.

### TGF-β mRNA transfection in human embryonic kidney 293 cell line

Human Embryonic Kidney (HEK) 293 cells were seeded into a 24-well cell culture plate at a density of 150,000 per well in Dulbecco’s Modified Enriched Medium (DMEM) containing fetal bovine serum (10%), L-glutamine, and penicillin-streptomycin. Cells were allowed to grow for 24 hours and then TGF-β mRNA (500 n per/well) was transfected into cells in triplicate using lipofectamine MessengerMax (Life Technologies, Carlsbad, CA) and OptiMEM serum free medium (ThermoFisher Scientific, # 31985–062, Rockford, IL) according to manufacturer guidelines. Cells were allowed to grow for an additional 24 hours and then samples collected in RIPA buffer (Sigma Aldrich, # R0278–500mL) containing cOmpleteMini protease cocktail inhibitors (Roche Diagnostics, # 11836153001). Samples were centrifuged (300 *g*, 5 min, 4°C) to remove cellular debris and supernatant was then collected. Protein concentrations were determined using a Pierce^™^ BCA Protein Assay (Peirce, # 23227).

### Immunoblots

Whole cell lysates samples (10 μg per well) were analyzed for TGF- β by denaturing SDS-PAGE 4–12% Bis-Tris followed by western blot analysis. Samples were transferred to PVDF membrane using an iBlot 2 dry apparatus (ThermoFisher Scientific, Rockford, IL), The membranes were blocked in non-fat dried milk (10% with 5% TTBS) and TGF- β was detected using primary anti TGF-β antibody (1:5,000, Abcam ab215715; Waltham, MA) overnight at 4°C, followed by a secondary goat anti-rabbit IgG HRP at 1:10,000 for 1 hour at room temperature. Membranes were stripped and re-probed with anti-GAPDH (Cell Signaling Technology, # 2118S, Danvers, MA) for 1 hour at room temperature at a 1:1,000 dilution, and a secondary goat anti-rabbit IgG HRP (Bio-Rad, # 170–6515 Bio-Rad, Hercules, CA) at a 1:10,000 for 1 hour at room temperature. Membranes were washed in ECL Prime Western Blotting Detection Reagent (Amersham Biosciences, Amersham, UK, # RPN2232) prior to visualization using an Amersham Cytiva imager (Amersham Biosciences, Amersham, UK).

To analyze TGF-β protein expression in lung tissue, immunoblots were performed on collected lung tissue, CD45 separated lung digest cells, or the large aggregate fraction from the bronchoalveolar lavage (BAL) fluid. Tissue from the accessory lobe was mechanically homogenized on dry ice and digested in lysis buffer with protease inhibitors (100 μL/0.33g tissue). The tissues were sonicated, centrifuged (2 min, 1,000 × *g*), and supernatants were assessed for protein concentration using a Pierce^™^ BCA Protein Assay. Equal amounts of protein from lung tissue samples (45 μg per well) were analyzed for TGF-β protein expression as described above. Antibodies for TGF-β were used at 1:5,000 and goat anti-rabbit HRP at 1:5,000. Membranes were washed in ECL Prime Western Blotting Detection Reagent prior to visualization on x-ray film.

### Co-assembly of IAJD34 and mRNA

IAJD34 was synthetized as previously described (28), and the purity and structural identity of final products and intermediates were determined using various techniques, including thin-layer chromatography (TLC), high-pressure liquid chromatography (HPLC), ^1^H and ^13^C NMR, and matrix assisted laser desorption ionization-time of flight (MALDI-TOF) mass spectrometry (28). Co-assembly of IAJD34 and mRNA was performed as previously described (28, 35). Briefly, nucleoside-modified mRNA encoding Luc mRNA or TGF-β mRNA was dissolved at a concentration of 4 mg/mL in UltraPure DNase/RNase-free PCR-certified water (Teknova, # W3440). IAJD34 was dissolved in ethanol at a concentration of 80 mg/mL. For the co-assembly of IAJD34 with mRNA, the mRNA was mixed with 10 mM acetate buffer (pH 4.0), and this solution was rapidly mixed with IAJD34 in ethanol at an IAJD34/mRNA weight-to-weight ratio of 40 and vortexed for 5 s. The prepared formulation was analyzed for size (nm) and polydispersity index (PDI) using dynamic light scattering (DLS) prior injection.

### Animal studies

Male and female wild-type BALB/c mice obtained from Charles River (Luc mRNA study) or Jackson Laboratories (TGF-β study) were used for all experiments. Mice were housed under standard conditions with food and water provided ad libitum. All experiments were conducted in accordance with the University of Pennsylvania and Rutgers University IACUC-approved protocols adhering to the U.S. National Institutes of Health Guide for the Care and Use of Laboratory Animals. Mice were housed and cared for in the Association for Assessment and Accreditation of Laboratory Animal Care International (AAALAC)-accredited facilities.

### Bioluminescence characterization for Luc mRNA delivery to mouse lungs

Female or male 6–8-week-old BALB/c mice were administered 10 μg of Luc-mRNA-IAJD34 in a100-μL volume via retro-orbital intravenous injection. Four hours post-injection, mice were injected intraperitoneally (i.p.) with D-luciferin (Regis Technologies) at a dose of 150 mg/kg of body weight. The mice were anesthetized in a ventilated anesthesia chamber with 3% isoflurane (Piramal Healthcare Limited) in oxygen and imaged 10 min post D-luciferin injection using an *in vivo* imaging system (IVIS, PerkinElmer, Waltham, MA). For organs imaging, mice were sacrificed, organs were collected immediately, and bioluminescence imaging was performed. Bioluminescence was quantified as proton flux (photons/seconds) in each region of interest using Living Image software (PerkinElmer) as previously described (35).

### TGF-β mRNA-IAJD34 formulation toxicity studies

TGF-β mRNA was co-assembled with IAJD34 as described above. The formulation was then concentrated using a Vivaspin ultrafiltration spin column MWCO 10,000 (Cytiva # 28–9322-47) according to manufacture guidelines. Mice were randomly assigned to control or treated. Mice were anesthetized with isoflurane and received a single retro orbital intravenous injection of 10 μg, 20 μg, or 30 μg of TGF-β mRNA co-assembled with IAJD34 or empty IAJD34. The following criteria to assess the toxicity of formulated TGF-β mRNA-IAJDs was utilized: mice behavior, serum and BAL fluid cytokine analysis, liver enzyme activities and main organs’ histological score. Mice were sacrificed 24 hours post-injections, and their serum samples and organs were collected. Organs were fixed in 4% paraformaldehyde, and further stained with H&E as described below. Liver, spleen, kidney, and lungs were inspected for signs of inflammation and deviations from normal histology.

### Enzyme-linked immunosorbent assay (ELISA)

ELISAs for aspartate aminotransferase (AST; Abcam ab263882) and alanine aminotransferase (ALT; Abcam ab282882) were performed in serum in a 1:80 dilution according to manufacture guidelines. Interleukin-6 (IL-6; Abcam ab222503) was used to measure serum and first wash BAL fluid levels in accordance with manufacturer guidelines.

### Multiplex cytokine analysis

Cytokine in the serum and first 1 mL of BAL fluid was performed by the University of Pennsylvania Human Immunology Core (RRID: SCR_022380) using a Milliplex Max Mouse Cytokine/Chemokine Magnetic Bead Panel - Premixed 32 Plex - Immunology Multiplex Assay (MCYTMAG-70K-PX32; MilliporeSigma, Burlington, MA). Samples were run in duplicate, and cytokine and chemokine concentrations were determined from a matched standard curve.

### Bleomycin mRNA-IAJD34 studies

Mice were randomly assigned to treatment groups. Mice were anesthetized with isoflurane and received a single retro orbital intravenous injection of 10 μg of TGF-β mRNA formulated with IAJD34 or empty IAJD34. while still under anesthesia, mice immediately received a single intratracheal instillation of either 50 μL PBS or 50 μL of bleomycin (3U/kg of body weight) (Santa Cruz Biotechnology, Inc., Dallas, TX; # sc-200134B) diluted in PBS as previously described (3). Following injections and treatment, mice were observed to ensure full recovery from anesthesia and that dose was successfully administered. Animals were weighed daily and provided supportive care when necessary. Mice were sacrificed 3 days post intratracheal administration of bleomycin via single intraperitoneal injection of ketamine (135 mg/kg of body weight) and xylazine (30 mg/kg of body weight) (Fort Dodge Animal Health, Fort Dodge, IA).

### Bronchoalveolar lavage

Lungs were lavaged with 1 mL of ice-cold PBS, followed by five 1 mL washes of ice-cold PBS through a 20-gauge canula inserted into the trachea. Collected BAL fluid was centrifuged at 300 × *g* for 8 mins. The cell-free supernatant from the first wash was collected for protein and cytokine analysis, and the cell-free supernatant from the five subsequent washes (5 mL) was collected for phospholipid analysis. Cell pellets from both washes were combined and resuspended in 1 mL of staining buffer (5% FBS in PBS, 0.2% sodium azide) and assessed for viability using Trypan Blue Solution (0.4%, ThermoFisher Scientific, Rockford, IL). Total cell count was determined using a Z1 Counter particle counter (Beckman Coulter). Approximately 10,000 cells were centrifuged on a Thermo Shandon Cytospin-3 at 750 rpm for 3 min onto a microscope slide, followed by Giemsa staining using a Hema 3^™^ Stat Pack (Fisherbrand, ThermoFisher Scientific, Rockford, IL). Total and differential cell counts were obtained. Cells were identified as macrophages, eosinophils, neutrophils, and lymphocytes by standard morphology.

### Phospholipids

Collected cell-free BAL fluid (5 mL) was centrifuged (20,000 × *g*, 4° C, 1hour) and separated into large and small aggregate surfactant fractions as previously described (3, 36). Phospholipids were extracted from the large aggregate fraction and resuspended in 30 μL 0.9% sodium chloride. Total phospholipids were measured by light absorbance at 830 nm using a standard curve ranging from 0 to 3.1 μg phosphate as previously described (3, 37) adapted from a previously published method (38).

### Lung tissue digest

Lung tissue from the right lobes was incubated at 37° C for 30 min with intermittent shaking in 5 mL of collagenase buffer (2 mg/mL collagenase type IV (Sigma Aldrich, St. Louis, MO) in RPMI 1640 (ThermoFisher Scientific, Rockford, IL) with 5% FBS (ThermoFisher Scientific, Rockford, IL)). The digested tissue was filtered through a 70 μm strainer, washed with RPMI with 5% FBS, and centrifuged (6 min, 400 × *g*). The cell pellet was lysed with Red Blood Cell Lysis Buffer (Sigma Aldrich, St. Louis, MO) for 5 min. The purified cell pellet was resuspended at a concentration of 1 × 10^8^ cells/mL PBS with 2% FBS and 1 mM EDTA. CD45 + leukocytes were isolated using the EasySep^™^ Mouse CD45 Positive Selection Kit (Stemcell Technologies, Cambridge, MA) and prepared for flow cytometry.

### Flow Cytometry

Cells from the BAL fluid or lung tissue digest were resuspended in 100 μL of staining buffer (PBS with 5% FBS, with 0.2% w/v sodium azide). Cells were incubated with TruStain FcX anti-mouse CD16/32 (Fc Block, 1:100) for 10 min at 4°C to prevent non-specific antibody binding. Cells were then incubated with antibodies against CD11b, CD206, CD11c, CD45, F4/80, Ly6c, MHC II, and Siglec Fat a 1:100 dilution in staining buffer (supplemental Table 1). After centrifugation and washing, cells were stained with eFluor 780-conjugated fixable viability dye for 30 min at 4°C, washed with staining buffer, and fixed in paraformaldehyde (3%). Fluorescence was analyzed using a Gallios 10-color flow cytometer (Beckman Coulter, Brea, CA). Cells were analyzed after sorting based upon forward and side scatter, doublet discrimination, and viability using Kaluza software (Beckman Coulter, Brea, CA). Discrete alveolar and interstitial macrophage phenotypes were determined as previously described (3).

### Seahorse real-time cellular metabolic analysis

Positively selected CD45 lung digest cells were plated at 200,000 cells per well in a poly-D-lysine coated Seahorse XF96 Cell Culture Microplate (Agilent, Santa Clara, CA) and incubated at 37°C, 5% CO_2_ for 1 hour to allow for cell adhesion. The cells were fed with ECAR medium (DMEM media, pH 7.4 (Agilent # 103575–100) with 2 mM L-glutamine) or OCR medium (DMEM media, pH 7.4 with 25 mM glucose, 1 mM pyruvate, and 2mM L-glutamine). The extracellular acidification rate (ECAR) and oxygen consumption rate (OCR) were measured using a Seahorse XF96 Analyzer (Agilent Technologies, Santa Clara, CA). For ECAR analysis, CD45 + cells were sequentially treated 25 mM glucose, 4 μM oligomycin, and 50 mM 2-deoxy-d-glucose (2-DG). For OCR measurement, the cells were sequentially treated with 4 μM oligomycin, 1 μM carbonyl cyanide ptrifluoromethoxyphenylhydrazone (FCCP), 0.5 μM rotenone, and 0.5 μM antimycin A. Data were normalized to μg of protein per well and analyzed using the Agilent Wave Software v. 2.6.1.

### Histology and immunohistochemistry (IHC)

After BAL fluid collection, the left lung lobe was inflation fixed in 3% of paraformaldehyde and embedded in paraffin. Liver, spleen, and kidney tissue were also fixed in 3% of paraformaldehyde and embedded in paraffin. Four-micrometer sections were cut, slide-mounted, and left unstained for IHC or stained with hematoxylin and eosin (H&E) to observe histological changes. For the TGF-β dose response toxicity study, tissues were blindly scored by a board-certified pathologist to determine overt toxicological pathology. For bleomycin studies, scans were blindly scored and quantified via ImageJ (NIH). For ImageJ quantification, samples were analyzed as previously described (3, 39). Briefly, randomly selected histological areas (n = 10, 400X) from each sample were captured and used to determine tissue consolidation (% white space), alveolar wall thickness, and cell infiltration (numbers of nuclei). Mounted unstained tissue sections were deparaffinized in xylenes followed by decreasing concentrations of ethanol (100 − 50%) and water. Antigen retrieval was performed in heated citrate buffer (10 mM sodium citrate, pH 6.0) for 30 min followed by quenching of endogenous peroxidase (3% H_2_O_2_ in methanol). Tissue sections were incubated in blocking buffer (10% normal goat serum in PBS) for 1 hour at ambient temperature to prevent non-specific binding. Tissues were incubated at 4°C for 18 hours with anti-firefly luciferase (Abcam ab238448; Waltham, MA 1:100) or TGF-β (Abcam ab215715; Waltham, MA 1:100) antibody in blocking buffer along with IgG controls (Pro-Sci 3703; Fort Collins, CO, matched concentrations). Sections were washed in decreasing concentrations of Tween-TBS (1%–0.5%) and incubated with biotin-conjugated secondary antibody (Vector Laboratories Vectastain Rabbit Kit; Newark, CA) for 1 hour at ambient temperature. Antibody binding was visualized beneath a microscope using a DAB Peroxidase Substrate Kit (Vector Laboratories, Newark, CA). Slides were scanned at 40X magnification using a VS120 Virtual Slide Microscope (Olympus, Waltham, MA) and viewed with OlyVIA software (Olympus, Waltham, MA) at 400X magnification.

### Statistical Analysis

Statistical analyses were completed using GraphPad Prism version 9 or 10. Results are reported as Means ± SEM unless otherwise indicated. Data were tested for normal distribution using a Shapiro-Wilks test. If normally distributed, statistical significance for multiple group comparisons was determined using a one-way ANOVA with Tukey’s post-hoc test or Šídák’s multiple comparison test. If not normally distributed, statistical significance for multiple comparisons was determined using a Kruskal-Walli’s test. For parametric single comparisons, statistical significance was determined using an unpaired t-test with Welch’s correction compared to control groups as indicated in figure legends. If single comparisons were nonparametric, statistical significance was determined using a Mann-Whitney U test. All P-values < 0.05 were considered statistically significant. For data presented with Data are presented as Median ± SE, statistical comparisons were made using a Wilcoxon ranked sum test. Statistical tests were conducted using a 5% significance level. n = 3–10 animals/group and is further indicated in figure legends.

## RESULTS AND DISCUSSION

### Characterization of lung-specific IAJD34 for targeted mRNA delivery to the lung

**Figure 1:**
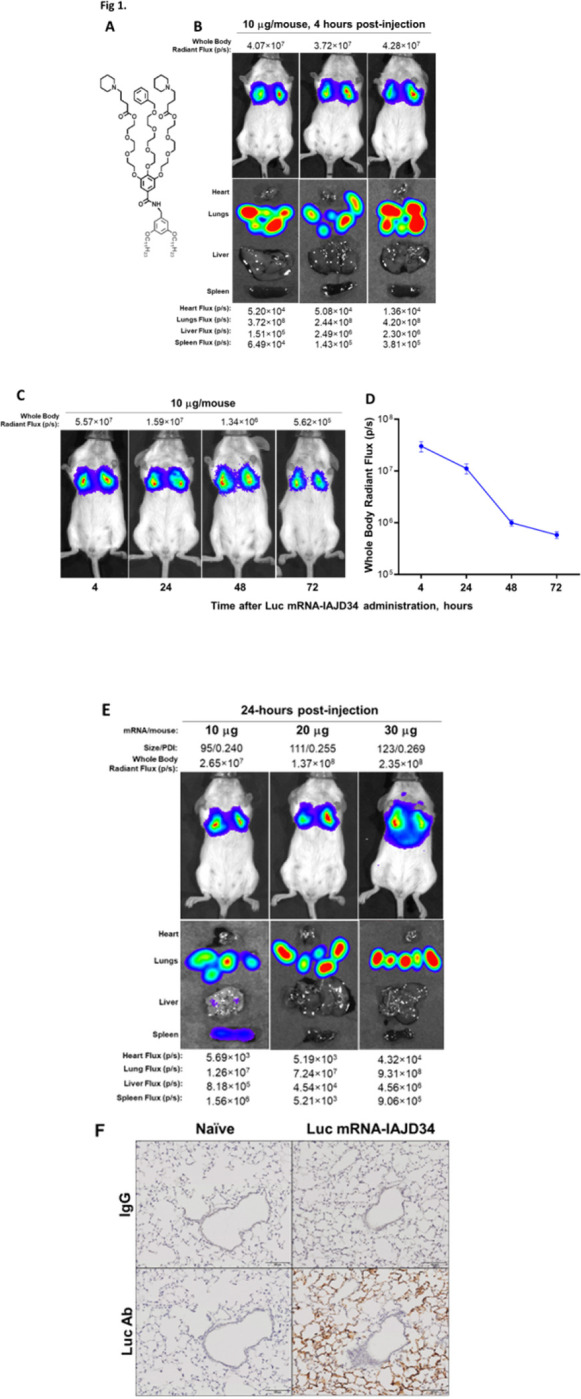
Specificity of IAJD34 for targeted mRNA delivery to the lung. IAJD34 formulated with luciferase mRNA (Luc mRNA-IAJD34) was i.v. injected into BALB/c mice and analyzed for efficient pulmonary mRNA using IVIS. **(A)** The chemical structure of one-component IAJD34 (40); **(B)** Specificity of Luc mRNA-IAJD34 delivery to the lung. Representative IVIS images of whole-body and organs of mice at 4 hours post-injection of 10 μg Luc mRNA-IAJD34 show luminescent biodistribution of luciferase expression predominantly in the lungs. IVIS images were analyzed, and the photon radiance (Lq) (s^−1^sr^−1^m^−2^) of each image was quantified. The quantitative bioluminescence imaging (BLI) data are shown as total flux (p/s); **(C)** Time course luciferase expression *in vivo*. Representative IVIS images of whole-body mice following 10 μg Luc mRNA-IAJD34, imaged at 4, 24, 48, or 72 hours post-injection; **(D)** Whole-body radiant flux (p/s) at various time points following the injection of 10 μg Luc mRNA-IAJD34. Data presented as mean+SEM, with n=12 for 4 hours, n=15 for 24 hours, n=18 for 48 hours, and n=6 for 72 hours. **(E)** Dose-dependent pulmonary delivery of Luc mRNA-IAJD34. Representative IVIS images of whole-body and organs of mice injected with Luc mRNA-IAJD34 at doses of 10, 20, or 30 μg, imaged at 24 hrs post-injection. Particle size (nm) and polydispersity index (PDI) for Luc mRNA-IAJD34 at each dose were measured prior injection and are shown above the corresponding mouse IVIS image; **(F)** Delivery of Luc mRNA-IAJD34 to lung parenchyma. At 24 hours post-injection with 30 μg Luc mRNA-IAJD34, lung tissue was harvested and IHC for luciferase or IgG was performed and compared to naïve animals.

Many IAJDs identified are capable of targeting the lung (40). With a relatively large chemical structure and a pKa of 6.74 lending to a strongly lung specific targeting, IAJD34 was selected for further characterization (Fig. 1A). To evaluate lung-specific delivery, the Luc mRNA-IAJD34 formulation was injected into mice at an initial dose of 10 μg per mouse. Four hours post-injection, whole-body and organ luminescence were quantified via IVIS. Luciferin intensity in the lungs was strong and several magnitudes higher compared to other organs (Fig. 1B). To evaluate the kinetics of luciferin protein production over time, mice were injected with the Luc mRNA-IAJD34 formulation at a dose of 10 μg per mouse. Live mice were imaged at various time points (4, 24, 48, and 72 hours) post-injection, and the luciferase intensity was quantified as a whole-body flux (p/s) (Fig. 1C). Luciferin intensity peaked at 4 hours post-injection (3.12 × 10^7^ ± 9.15 × 10^6^), by 24 hours, the flux intensity decreased but remained elevated by 37%. At 48 and 72-hours post-injection, there was a more significant drop to 3% and 1.9%, respectively (Fig. 1D, Supplemental Table 2).

The stability of the Luc mRNA-IAJD97 formulation was studied over time at + 4°C. The formulation was stored at + 4°C for 5 days and then injected into mice. Whole-body luciferase intensity did not change significantly compared to the freshly prepared formulation (Supplemental Fig. 1). Thus, the one-component IAJD34 formulated with Luc mRNA maintains stability and activity at 4°C and can be stored for at least 5 days prior to use.

To evaluate the impact of dose on luciferase expression, Luc mRNA-IAJD34 was injected at 10, 20, and 30 μg doses per mouse and imaged at 24 hours post-injection. There was a dose-dependent increase in lung-specific luciferase expression, reaching 9.3 × 10^8^ at 30 μg per mouse (Fig. 1E, Supplemental Table 3). Immunohistochemical staining of lung tissue with the 30 μg dose revealed that expression of luciferase protein was well distributed throughout the alveolar epithelium, with minimal staining in the upper airway compared to the control (Fig. 1F). The mRNA delivery to the alveolar level (i.e., below the 16th generation of the airway tree) had previously been unsuccessful, the expression of luciferase in these lower respiratory zones suggests our approach may serve as a promising therapeutic.

Notably, our evaluation of IAJD34 toxicity and efficacy was limited using this formulation because luciferase is encoded by a large mRNA and has no biological function within the cell. Thus, delivery of clinically relevant mRNA was essential to fully characterize the therapeutic nature of IAJD34.

### Evaluation the pulmonary delivery of therapeutic mRNA

Following confirmation of IAJD34 lung specificity, we focused on designing a therapeutic mRNA for lung-targeted delivery. TGF-β, a prominent anti-inflammatory cytokine associated with the resolution of ALI, was selected as our mRNA of interest (30, 41). The production and quality of TGF-β mRNA were validated (Supplemental Fig. 2A). Protein expression of TGF-β was confirmed in HEK293 cells following transfection with TGF-β mRNA (Supplemental Fig. 2B).

**Figure 2:**
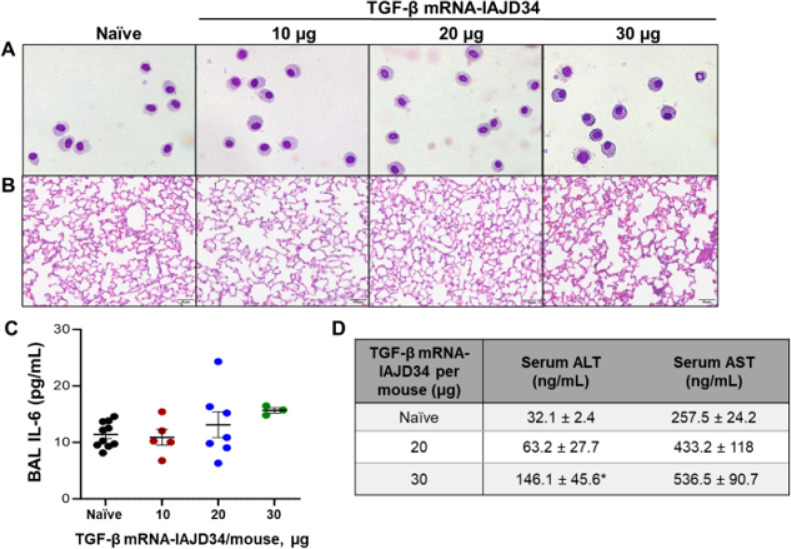
Evaluation of dose-dependent toxicity of TGFβ mRNA delivery with IAJD34. Various doses of TGFβ mRNA-IAJD34 (10, 20, and 30 μg per mouse) were delivered to BALB/c mice and compared to naïve animals. At 24 hours post-injection, BAL fluid and cells, whole lung tissue, and serum were collected. **(A)** BAL cells were fixed and stained using a Hema 3 Stat kit. Representative H&E images are shown; **(B)** Formaldehyde-fixed lung tissue was processed, stained with H&E and scored by a board-certified pathologist. Representative H&E images are shown; **(C)** IL-6 was measured in BAL fluid via ELISA according to manufacturer’s guidelines; **(D)** ALT and AST were measured in serum via ELSIA according to manufacturer’s guidelines. Data are presented as mean ± SE, n=3–10/group from 2 independent experiments. For C and D, samples were compared using Shapiro-Wilk test for normality followed by an ordinary one-way ANOVA with Tukey’s Multiple comparison where *indicates a significant difference from the naïve control, p<0.05.

To evaluate inflammatory effects in the lung, IAJD34 formulated with TGF-β mRNA (TGF-β mRNA-IAJD34) was injected at 10, 20, and 30 μg per mouse and BAL markers were measured 24 hours post-injection. Increasing doses (10 μg and 20 μg) had no effect on BAL phospholipids or total cell count in the BAL fluid (Table 1). However, a significant increase in BAL fluid protein and neutrophils was observed at the 30-μg dose, indicating epithelial barrier dysfunction (Table 1, Fig. 2A). Although BAL fluid protein was increased, there was no significant increase in BAL IL-6 levels (Fig. 2C), suggesting this may have been a downstream effect of abundant TGF-β delivery rather than a side effect of IAJD34 formulation.

Histological sections of organs were stained with H&E and evaluated by a board-certified pathologist. No pathological alterations were observed in the liver, spleen, or kidney at any of the tested doses (Supplemental Fig. 3). In the lung histology, a dose-dependent increase in fibrin deposition and lymphocyte infiltration was observed, as was expected at higher doses of TGF-β (Fig. 2B). Liver function was also evaluated by measuring serum aspartate aminotransferase (AST) and alanine aminotransferase (ALT), known markers of liver function (42). Increased ALT was observed at the 30 μg dose, with no significant change in AST (Fig. 2D). Thus, systemic pathological and toxicological markers are limited in the 10 μg and 20 μg doses of TGF-β mRNA-IAJD34. Even at the highest dose, 30 μg, increases in systemic toxicity markers were minimal. Established four-component LNPs are typically dosed between 1 and 10 μg for vaccinations for SARS-COV2 in mice, indicating that 10 μg would likely be sufficient (43). Additionally, most studies using IAJDs report that 10 μg or less of mRNA to be effective for organ-specific detection (40).

**Figure 3:**
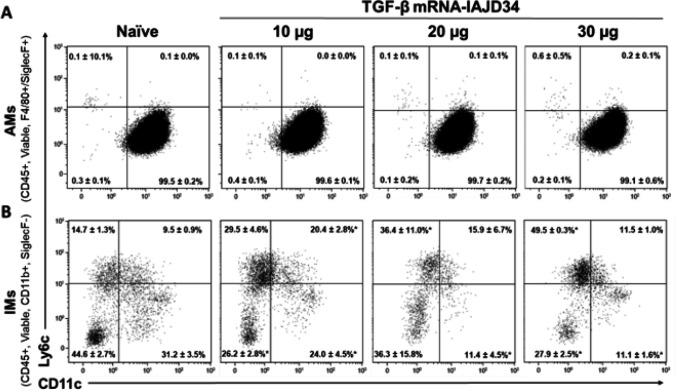
Absence of inflammatory activation in response to increasing doses of TGF-β mRNA-IAJD34 in alveolar macrophages and interstitial macrophages. Various doses of TGFβ mRNA-IAJD34 (10, 20, and 30 μg per mouse) were delivered to BALB/c mice and compared to naïve animals at 24 hours post-injection. **(A)**Cells from the BAL were isolated, immunostained, and analyzed by flowcytometry. Cells that were positively stained for both Siglec F and F4/80 were determined to be alveolar macrophages (AMs) and were evaluated for Ly6c and CD11c expression; **(B)** Cells from digested lung tissue were immunomagnetically separated based upon CD45 expression. CD45+ cells were isolated, immunostained, and analyzed. Cells that expressed F4/80 and CD11b in the absence of Siglec F were categorized as interstitial macrophages (IMs) and were analyzed for Ly6c and CD206 expression. Data are presented as mean ± SE for n=4–10 per group. Samples were compared using Shapiro-Wilk test for normality followed by an ordinary one-way ANOVA with Šídák’s multiple comparisons test where * indicates a significant difference from the naïve control, p<0.05.

To determine alveolar macrophage (AM) activation, BAL cells were evaluated by flow cytometry as previously described (3). AMs were defined as viable CD45^+^F4/80^+^SiglecF^+^ cells (Supplemental Fig. 4). Inflammatory activation was characterized by expression of Ly6c and CD11c. No dose-dependent difference was observed in AM phenotype, with a majority of cells being resident AMs (Ly6c−/CD11c+) under all conditions. This was consistent with the lack of observed acute inflammatory activation (Fig. 3A). There were also no differences in Ly6C/CD206 AM populations at any dose of TGF-β mRNA-IAJD34 compared to control (Supplemental Table 4). Interstitial macrophages (IMs) were identified in CD45 + lung digest cells. IMs were defined as viable being CD45^+^, CD11b^+^, SiglecF^−^ cells as previously described (Supplemental Fig. 4) (3). Ly6c^+^ IMs were increased in all doses of TGF-β mRNA-IAJD34 with no increases in either CD11c^+^ or CD206^+^ expression at any dose (Fig. 3B, Supplemental Fig. 5). Ultimately, no acute inflammatory activation was observed in IMs as a result of TGF-β mRNA-IAJD34 treatment when compared to control. It is unclear why treatment with TGF-β mRNA-IAJD34 increased Ly6c in IMs, but a previous study evaluating renal interstitial macrophages in Tgfbr2^fl/fl^ mice showed a decrease in Ly6c^+^ macrophages indicating that there may be a relationship between Ly6c and TGF-β signaling (44). Further analysis will be necessary to fully understand TGF-β mRNA-IAJD34’s effects on Ly6c^+^ IMs.

**Figure 4:**
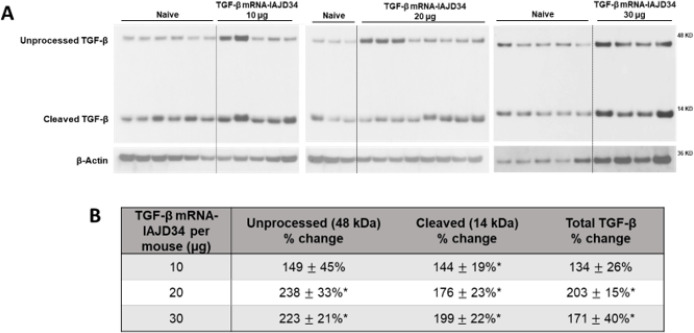
Dose-dependent expression of TGF-β mRNA-IAJD34 in lung tissue. Various doses of TGF-β mRNA-IAJD34 (10, 20, and 30 μg per mouse) were delivered to BALB/c mice and lung tissue was collected 24 hours post-injection and analyzed for TGF-β protein expression. **(A)** Western blots for TGF-β were performed on homogenized whole lung tissue at indicated time points. Representative images are shown; **(B)** Western blots results were quantified using densitometry. TGF-β protein expression was normalized to β-actin for each band, and the density was expressed relative to the naïve control. Data are presented as mean ± SE for n=4–9 per group. Statistical comparisons were made using a Mann-Whitney U test where * indicates a significant difference from control, p<0.05.

TGF-β protein exists in an unprocessed form with a latency-associated peptide that is cleaved to form an active protein, which can then initiate signal transduction (45, 46). To confirm that TGF-β mRNA-IAJD34 was reaching the lung and being processed, TGF-β protein expression was confirmed in lung tissue digest using western blotting. Cleaved TGF-β was significantly increased at all doses compared to the control, whereas unprocessed and total TGF-β were only significantly increased in the 20 μg and 30 μg groups (Fig. 4).

**Figure 5:**
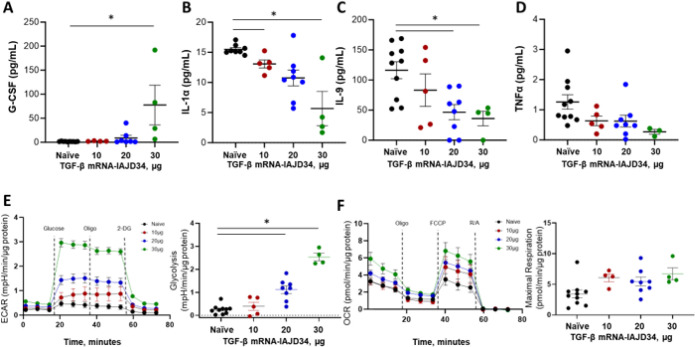
TGF-β mRNA-IAJD34-induced alterations in BAL cytokine production and cellular metabolic function. Various doses of TGF-β mRNA-IAJD34 (10, 20, and 30 μg per mouse) were delivered to BALB/c mice and BAL fluid was collected 24 hrs post-injection. **(A-D)** Cell- free BAL was evaluated for cytokines using a Milliplex Max Mouse Cytokine/Chemokine Magnetic Bead Panel - Premixed 32 Plex – Immunology Multiplex Assay. The concentration of **(A)** G-SCF, **(B)** IL-1α, **(C)** IL-9, and **(D)** TNFα are presented as pg/mL; **(E-F)** Cells from digested lung tissue were immunomagnetically separated based upon CD45 expression. CD45+ cells were isolated and analyzed metabolically using an Agilent Seahorse according to manufacturer’s guidelines; **(E)** ECAR was measured following injections of glucose, oligomycin (Oligo), and 2-DG, and glycolysis was determined following injection of glucose; **(F)** OCR was measured with injections of oligo, FCCP, and rotenone/antimycin (R/A) and normalized to non-mitochondrial energy production. Maximal respiration was determined following FCCP injection. Data are shown as mean ± SE, n = 4–10 per group. Data were evaluated for normality using a Shapiro- Wilk’s test and compared using a one-way ANOVA followed by Tukey’s multiple comparisons test. * indicates a significant difference from the control, p<0.05.

We next evaluated the presence of other cytokines and chemokines in the BAL fluid that can be upregulated or downregulated by TGF-β signaling (Supplemental Table 5). Granulocyte-colony stimulating factor (G-CSF), identified as an anti-inflammatory pro-neutrophilic growth factor, was elevated at the 30 μg dose of TGF-β mRNA-IAJD34 compared to the control (Fig. 5A) (47). By contrast, pro-inflammatory cytokines interleukin-1 alpha (IL-1a), interleukin-9 (IL-9), and tumor necrosis factor (TNFα) were decreased in a dose-dependent manner (Fig. 5B–D) (48, 49). Thus, at 24 hours post-treatment, TGF-β protein expression alters the downstream signaling of pro- and anti-inflammatory cytokines. These observations are likely directly associated with increased TGF-β signaling and not an off-target effect of IAJD34.

Finally, we studied metabolic upregulation, which is predicted to occur with increased protein synthesis and cell signaling stimulated by TGF-β mRNA. CD45^+^ lung-digest cells were evaluated by employing the glycolytic rate assay and the mito stress. For the glycolytic rate assay, extracellular acidification rate (ECAR) was recorded. Glycolysis was determined by the ECAR measurement prior to glucose injection subtracted from the maximum ECAR before oligomycin injections. Injection with TGF-β mRNA-IAJD34 caused a dose-dependent increase in glycolysis, with a significant increase observed in the 20 μg and 30 μg groups compared to the control (Fig. 5E). This increase was associated with increased cytokine production, likely a direct impact of TGF-β signaling cascades (50, 51). For the mito stress test, oxygen consumption rate (OCR) was used to determine oxidative phosphorylation. OCR data were first normalized to the post rotenone/antimycin (R/A) injections to control for non-mitochondrial oxygen consumption. Maximal respiration was determined from OCR data as the maximum OCR following the injection of FCCP (Carbonyl cyanide-p-trifluoromethoxyphenylhydrazone), a potent mitochondrial uncoupler. An TGF-β mRNA-IAJD34 induced dose-dependent increase was observed in maximal respiration, though this was not significant (Fig. 5F). Although increased metabolic demand is likely a part of increased translational processes, it has also been hypothesized to be a direct effect of increased TGF-β expression and macrophage activation (51). Thus, treatment with TGF-β mRNA-IAJD34 successfully alters macrophage cell phenotype and cytokine expression as expected with the production of TGF-β protein.

### Evaluating pulmonary delivery of TGF-β mRNA-IAJD34 over

Due to the lack of toxicity, limited alterations in cell phenotype, and efficient expression of TGF-β protein and downstream signals, the 10 μg dose of TGF-β mRNA-IAJD34 was selected for further development.

The 10 μg dose of empty-IAJD34 or TGF-β mRNA-IAJD34 was further evaluated at 4, 24, and 48 hours post-injection. Treatment with TGF-β mRNA-IAJD34 did not increase BAL protein or phospholipids at any time point (Supplemental Fig. 6).

**Figure 6:**
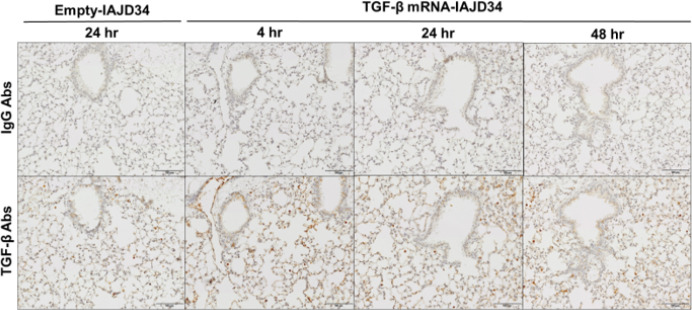
Cell specific expression of TGF-β mRNA-IAJD34 in the lung over time. Empty IAJD34 or 10 μg TGF-β mRNA-IAJD34 were delivered to BALB/c mice, and lung tissues were evaluated for TGF-β protein expression at 4, 24, and 48 hrs post-injection. Lung tissues was stained for TGF-β or IgG control via immunohistochemistry. Representative IHC images are shown. The slides are at 100x magnification, and the scale bars presented 100 μm. Increases in TGF-β protein expression were observed in the TGF-β mRNA-IAJD34 group through immunohistochemical staining from 4 to 48 hours.

**Figure 7:**
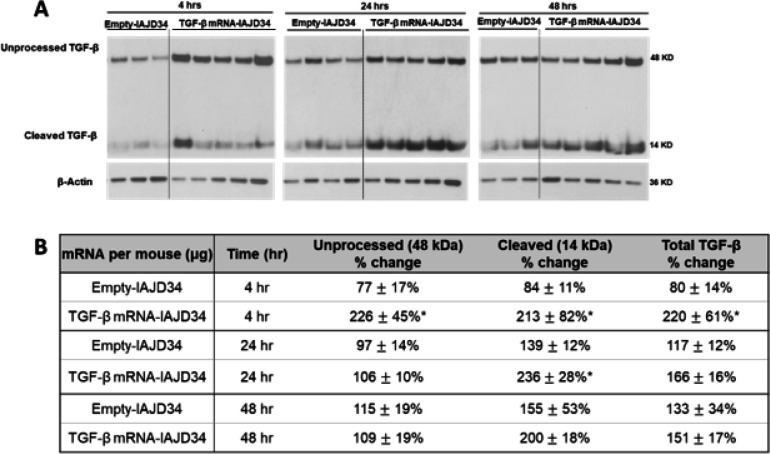
TGF-β protein expression is transiently induced after mRNA delivery to lung via IAJD34. Empty IAJD34 or 10 ug TGF-β mRNA-IAJD34 were delivered to BALB/c mice and lung tissue homogenates were evaluated for TGF-β protein expression at 4, 24, and 48 hr post-injection. **(A)**Western blots for TGF-β were performed on homogenized whole lung tissue. Representative images are shown. **(B)**Western blots bands were quantified by densitometry and TGF-β protein expression was normalized to β-actin for each band, and the density was expressed relative to the naïve control. Data are presented as mean ± SE, n = 3–5 per group. Statistical comparisons were made using a Mann-Whitney U test, where * indicates a significant difference from the matched empty control, p<0.05.

IHC staining of lung tissue confirmed that TGF-β protein expression was transiently expressed in lung tissue, peaking at 4 hours post-injection, and persisting up to 48 hours (Fig. 6). Unprocessed and cleaved TGF-β were also measured in whole lung tissue, showing significant increases in all forms of TGF-β at 4 hours post-injection, an increase in cleaved TGF-β at 24 hours, and no significant increases in any TGF-β isoforms at 48 hours post-injection compared to the control (Fig. 7). Consistent with TGF-β dose-response findings, there were no changes in AM or IM inflammatory activation at any time point compared to control, though slight increases in Ly6c + IMs were observed (Supplemental Fig. 7 and Supplemental Table 6). Thus, a 10-μg dose of TGF-β mRNA-IAJD34 can deliver robust and transient TGF-β to the lung without significant signs of inflammation or toxicity. Transient expression is therapeutically beneficial as prolonged TGF-β expression can lead to fibrin deposition and fibrosis (29). Additionally, controlled repeat doses of TGF-β mRNA-IAJD34 can be delivered until resolution is obtained with a lower risk of over expressing TGF-β.

### TGF-β mRNA-IAJD34 can limit pulmonary injury following exposure to bleomycin

The ultimate goal of lung-targeted delivery of therapeutic mRNA is to generate treatment strategies for pulmonary injury and disease. Intratracheal bleomycin (ITB) is a laboratory model of ALI, characterized by acute pulmonary inflammation occurring over the first 7 days, transitioning to fibrotic development around 14 days, and resolving at 21–28 days post initial exposure (3, 4, 52). It should be noted that not all models of ITB result in the same level of fibrosis (53–55). To have interventive therapeutic potential, treatment with TGF-β is predicted to be most effective during the development of pro-inflammatory cellular activation and pathology.

For initial studies, animals were treated with a 10-μg dose of TGF-β mRNA-IAJD34 or empty-IAJD34 concurrently to instillation of bleomycin or PBS control. To align with initial pulmonary inflammation development and the expression of TGF-β mRNA-IAJD34, animals were euthanized 3 days post ITB or PBS exposure. ITB causes weight loss in mice over the course of the first 7 days (54). Substantial weight loss was observed in groups exposed to ITB, where treatment with TGF-β mRNA-IAJD34 had no impact on ITB-induced decreases in percent body weight (Supplemental Fig. 8).

**Figure 8:**
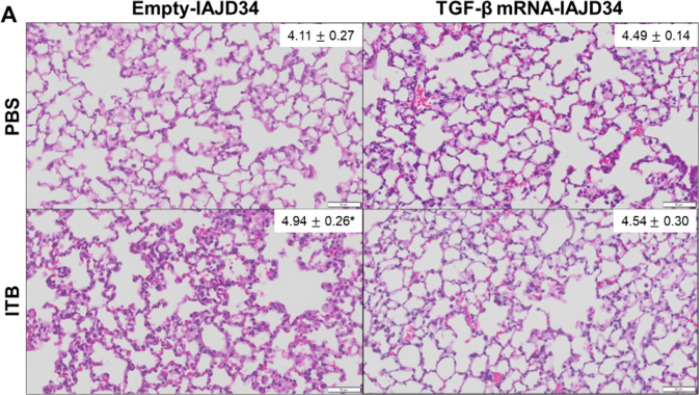
Effects of TGF-β mRNA-IAJD34 on bleomycin-induced histological alterations. BALB/c mice were exposed to PBS or bleomycin (ITB) and subsequently received either an empty IAJD34 or 10 μg TGF-β mRNA-IAJD34. Lung tissue was collected 3 days post exposure and injection. Fixed lung tissue was processed and stained with H&E. Representative H&E images are shown. The slides are shown at 400x magnification, with scale bars indicating 50 μm. An inset image shows the data of evaluating alveolar thickness, where 10 fields per slide were randomly selected, and analyzed using ImageJ. Data are presented as Median ± SE, n = 6–10 per group. Statistical comparisons were made using a Wilcoxon ranked sum test, where * indicates a significant difference from the PBS/empty-IAJD34 group, p<0.05.

ITB is also associated with increases in alveolar thickness, immune cell infiltration, and tissue consolidation that variably develop over the first 7 days post-exposure (3, 4, 39, 52). An increase in alveolar epithelial thickness was observed in ITB + empty-IAJD34 animals, with limited changes in percent white space and nuclei count compared to control (Fig. 8). Treatment with TGF-β mRNA-IAJD34 did not significantly increase epithelial thickness compared to the matched control, indicating TGF-β mRNA-IAJD34 can prevent some ITB-induced increases in lung pathology (Fig. 8). The limited ITB-induced histological alterations are likely a consequence of evaluating injury at such an early timepoint and a limitation of using BALB/c mice as a model. Notably, BALB/c mice are slightly more resistant to ITB exposure as compared to C57BL6/J mice, though the exact reasons for this are not well understood (53–56). BALB/c mice were chosen for this study as IAJD targeting and development has primarily been characterized in this strain of mice (28, 35, 40).

**Figure 9:**
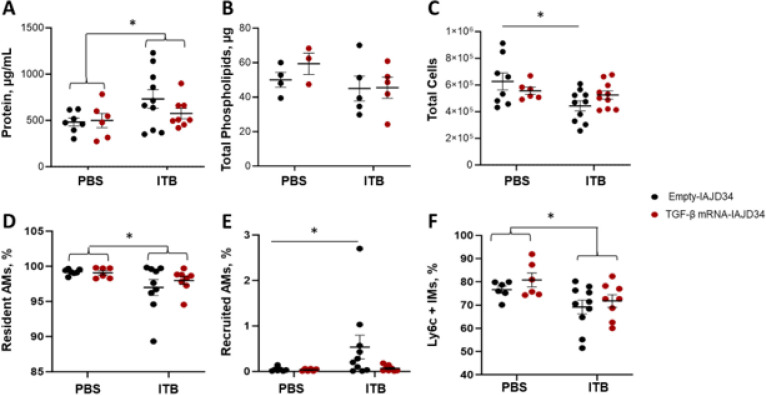
Effects of TGF-β mRNA-IAJD34 bleomycin-induced injury. BALB/c mice were exposed to PBS or bleomycin (ITB) and subsequently received either an empty IAJD34 or 10 μg TGF-β mRNA-IAJD34. Cell-free BAL fluid, BAL cells, and large aggregate surfactant fractions were collected 3 days post exposure and injection. **(A)** Cell-free BAL fluid was evaluated for total protein content using a BCA assay. Data were evaluated for normality using a Shaprio-Wilks normality test. PBS and Bleo were compared using a 2-way ANOVA; **(B):** Total phospholipids were determined from the large aggregate surfactant fraction. Data were compared using a 2-way ANOVA; **(C):** Total BAL cells were counted using a coulter counter. Data were compared using a 2-way ANOVA with Šídák’s multiple comparisons test; **(D-E):** BAL cells were immunostained for flow cytometric analysis. Cells that were positively stained for both Siglec F and F4/80 were determined to be alveolar macrophages (AMs). Resident macrophages (CD11c+/CD11b−), can be differentiated from recruited (CD11c−/CD11b+) or migratory macrophages (CD11c+/CD11b+). Resident AMs exposed to PBS and ITB were compared using a 2-way ANOVA. Recruited AMs were compared using a Wilcoxon Signed Rank test. **(F)** Cells from digested lung tissue were immunomagnetically separated based upon CD45 expression. CD45+ cells were isolated, immunostained, and analyzed. Cells that expressed F4/80 and CD11b in the absence of Siglec F were categorized as interstitial macrophages (IMs) and were analyzed for Ly6c expression. Ly6c+ IMs exposed to PBS and ITB were compared using a 2-way ANOVA. All data were evaluated for normality using a Shaprio-Wilks normality test and are presented as Mean ± SE, n = 3–10/group. * indicates a significant difference from the control, p<0.05.

Beyond histopathological alterations, ITB-induced ALI is also associated with increases in pulmonary epithelial injury, marked by increased BAL fluid protein and disrupted epithelial lipid barriers (57). Exposure to ITB caused a significant increase in BAL fluid protein content as compared to control, with TGF-β mRNA-IAJD34 treatment having no effect (Fig. 9A). Neither ITB nor TGF-β mRNA-IAJD34 had an effect on BAL fluid phospholipid levels, hypothesized to be a limitation of the 3-day time-point (Fig. 9B). However, treatment with TGF-β mRNA-IAJD34 prevented ITB-induced loss of cells lining the airway, a consequence of early pulmonary inflammation and cell death (Fig. 9C) (3).

To characterize the loss of BAL cells, AMs (viable CD45^+/^Siglec F^+/^F4/80^+^ BAL cells) were characterized further as being resident (CD11c^+^CD11b^−^), recruited (CD11c^−^CD11b^+^), or migratory macrophages (CD11c^+^CD11b^+^). In line with previous 7-day studies, exposure to bleomycin reduced resident AMs and increased recruited AMs compared to PBS control (Fig. 9D–E) (3, 4). Although treatment with TGF-β mRNA-IAJD34 did not prevent ITB-induced decreases in resident AMs, treatment did mitigate ITB-induced increases in recruited AMs (Fig. 9D–E). In contrast to TGF-β mRNA-IAJD34 dose response studies, Ly6c^+^ IMs (viable CD45^+^CD11b^+^SiglecF^−^ lung-digest cells) were decreased in ITB exposed animals irrespective of TGF-β mRNA-IAJD34 treatment (Fig. 9F). ITB-induced changes in AM and IM populations were variable between animals due to the early inflammatory time point and the less sensitive BALB/c model. Treatment with TGF-β mRNA-IAJD34 helped mitigate some AM alterations but had no effect on IMs at this time point. Innate TGF-β expression is expressed more highly in human AMs when compared to other lung cell types (58). There is some evidence that TGF-β plays less significant role in IM cell maturation and activity, but this remains largely speculative (58). Further model development will be necessary to understand the role that TGF-β plays in modulating ITB-induced AM and IM cell characteristics.

**Figure 10:**
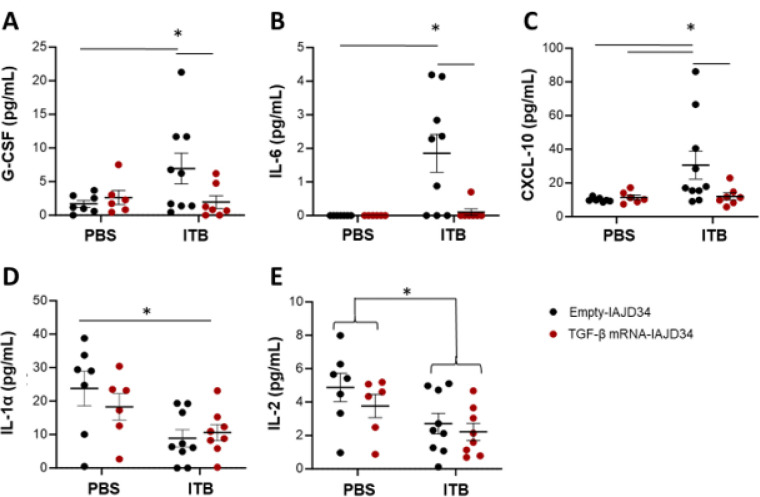
Effects of TGF-β mRNA-IAJD34 bleomycin-induced cytokine production. BALB/c mice were exposed to PBS or bleomycin (ITB) and subsequently received either an empty IAJD34 or 10 μg TGF-β mRNA-IAJD34. Cell-free BAL fluid was collected 3 days post exposure and injection. **(A-E)** Cell-free BAL fluid was evaluated for cytokines using a Milliplex Max Mouse Cytokine/Chemokine Magnetic Bead Panel - Premixed 32 Plex - Immunology Multiplex Assay. The concentration of **(A)**G-SCF, **(B)** IL-6, **(C)** XCL10, **(D)** IL-1α, and (E) IL-2 are presented as pg/mL. Date shown a mean ± SE, n = 6–10 per group. Data were evaluated for normality using a Shapiro Wilk’s test. If data were normally distributed, they were compared using a 2-way ANOVA followed by Šídák’s multiple comparisons test. If data were not normally distributed, they were evaluated using a Kruskal Wallis test. * indicates a significant difference, p<0.05.

We next evaluated the downstream effects of TGF-β mRNA-IAJD34 on cytokine signaling following exposure to ITB beyond pathological and inflammatory indicators (Fig. 10, Supplemental Table 7). As anticipated, exposure to ITB elevated levels of G-CSF, IL-6, and CXCL-10 compared to control. These neutrophilic or pro-inflammatory cytokines are elevated following ITB exposure (Fig. 10A–C) (59–62). Treatment with TGF-β mRNA-IAJD34 prevented ITB-induced increases in all three of these proinflammatory cytokines, consistent with the anti-inflammatory signaling of TGF-β (Fig. 10A–C). Exposure to ITB caused a reduction in IL-1α and IL-2 compared to PBS control, irrespective of treatment with TGF-β mRNA-IAJD34 (Fig. 10D–E). Both IL-1α and IL-2 are also pro-inflammatory cytokines, and it is unclear why ITB exposure decreased these cytokines, though it may be a result of AM resident cell loss. Thus, treatment with TGF-β mRNA-IAJD34 can prevent components of ITB-induced pulmonary inflammatory signaling.

## Conclusion

IAJD34 is a single-component lipid particle designed for the targeted delivery of mRNA to the lung (40). The current studies have shown that IAJD34 can successfully deliver luciferase mRNA to the lung, specifically targeting lung parenchyma. This is of particular therapeutic advantage as targeting the smaller airways, especially the respiratory regions beyond the 16^th^ generation of the respiratory tree, is necessary to treat most lung injury and consequent disease (*1, 5, 7*). While necessary, targeting these areas of the lung is challenging and has proven difficult in clinical settings (1).

We hypothesize that IAJD34 is able to target these areas due to the large and charged altering ester and amide groups in the head group. When injected intravenously, the large charge density traps the formulated IAJD34 in narrow vascular regions of the lung, ultimately delivering the mRNA of interest to the small pulmonary arteries. TGF-β mRNA-IAJD34 was able to deliver dose-dependent levels of TGF-β mRNA to the lung with limited inflammation and toxicity observed. Once delivered, TGF-β protein was produced, processed, and mediated downstream cytokine signaling. Delivery of TGF-β was transiently expressed over the course of 48 hours, which is important as long-term delivery of TGF-b can lead to significant fibrosis.

TGF-β mRNA-IAJD34 was used to treat ITB effects on the lung 3 days post-injury showed only mild improvements in lung histology and barrier function. Although the use of the day 3 timepoint in BALB/c mice meant that these factors were only mildly affected by ITB, our intention was to demonstrate that delivery of TGF-β mRNA-IAJD34 to the lung would reduce inflammatory signaling. Indeed, there was a significant TGF-β mRNA-IAJD34 effect that correlated with TGF-b expression. These results indicate that we could successfully deliver TGF-b to the lower lung and observe a significant signaling effect. Furthermore, this effect appears to be transitory in nature as would be required in a therapeutic setting.

To establish TGF-β mRNA-IAJD34 as a therapeutic in this ALI model, we will need to characterize the effect of TGF-b at 7 days or longer post-injury, which may require multiple dosing regimens. This study establishes the potential use of IAJD34 to treat ALI and other pulmonary ailments that currently require targeted clinical interventions and demonstrate the potential of mRNA delivery for therapeutic use in the lung.

## Figures and Tables

**Table 1.** Inflammatory response to TGF-β mRNA-IAJD34 pulmonary delivery.

TGF-β mRNA-IAJD34 per mouse (mq)	BAL Phospholipids (mq)	BAL Protein (mg/mL)	BAL Cell Count (×10^4^)			
			Total	MP	NP	LP
Naïve	40.2 ± 17.5	0.23 ± 0.02	26.4 ± 8.3	25.5 ± 7.8	0.2 ± 0.2	0.7 ± 0.4
10	53.1 ± 12.4	0.40 ± 0.27	17.6 ± 11.0	20.2 ± 6.9	0.1 ± 0.2	0.8 ± 0.7
20	42.4 ± 19.2	0.36 ± 0.18	29.9 ± 12.6	28.3 ± 11.8	0.9 ± 1.3	0.8 ± 0.5
30	60.2 ± 15.4	1.25 ± 0.76*	39.8 ± 2.47	35.5 ± 23.6	25.0 ± 25.2*	1.8 ± 1.6

IAJD34 loaded with TGF-β mRNA at doses of 10, 20, and 30 μg per mouse were delivered to BALB/c mice and compared to naïve animals. At 24 hours post-injection, total BAL protein, differential BAL cell count, and BAL phospholipid levels were quantified as markers of lung injury: MP, Macrophage, NP, neutrophil, LP, Lymphocyte. Data are presented as mean ± SD, n=3–10 per group. Samples were compared using Shapiro-Wilk test for normality followed by an ordinary one-way ANOVA with Tukey’s Multiple comparison where * indicates a significant difference from the control, p<0.05.
